# Supplementary material for: Getting to the Core of the Matter—Assessing the Role of Replication in Metabarcoding‐Based sedaDNA
Source: Mol Ecol Resour. 2026 Sep 3;26(7):e70200. doi: 10.1111/1755-0998.70200 (PMC13542785; doi:10.1111/1755-0998.70200)
Supplement: Supplementary file 1 [file MEN-26-e70200-s004.docx]

**Supplementary material**

**Supplementary data**

**Supplementary data 1:** Radiometric dating data, including raw ^²¹⁰^Pb measurements and age-depth model outputs (CF:CS model).

**Supplementary data 2:** X-ray fluorescence (XRF) data, including raw elemental counts and normalised geochemical profiles.

**Supplementary data 3:** Full statistical outputs from hierarchical modelling of species communities (jSDM), including model diagnostics and variance partitioning results.

**Supplementary figures**

**Fig. S1:** ^210^Pb activity-depth profiles for sediment cores from (A) Santoña (STO), (B) Cicero (CCO), and (C) Colindres (CLR). ^210^Pb activities are shown against the upper x-axis, while the natural logarithm of excess or unsupported ^210^Pb is shown against the lower x-axis. Points with black outlines represent ln-transformed excess ^210^Pb values used to estimate sediment accumulation rates using the “Simple” Model (Robbins, 1978, dotted regression lines). Error bars represent analytical uncertainty. In the STO core, excess ^210^Pb activity is confined to the upper ~10 cm, indicating a highly condensed sediment sequence.

**Fig. S2:** Standardised geochemical XRF profiles across biological replicates (cores) at sites CCO and CLR. Elemental concentrations (Fe, Rb, Ti and Zr) are shown as depth-standardised z-scores to facilitate comparison among cores. Lines represent individual biological replicates and illustrate closely matching stratigraphic trends across spatially separated cores within each site.

**Fig. S3:** Rarefaction and coverage-based diversity estimates for metabarcoding-based *seda*DNA datasets. Panels (A) and (B) show sample-size-based rarefaction and extrapolation curves for the COI and 18S markers, respectively. Solid lines represent interpolation and dashed lines extrapolation, with shaded areas indicating 95% confidence intervals. Panels (C) and (D) show coverage-based rarefaction and extrapolation curves derived from iNEXT for COI and 18S, respectively, illustrating sample completeness across sites.

**Fig. S4:** Relative abundance of metazoan phyla detected across the three intertidal sites (STO, CCO and CLR) based on metabarcoding-based *seda*DNA. Bars represent the mean relative abundance of amplicon sequence variants (ASVs) per core after collapsing eight technical PCR replicates. Facets indicate genetic markers (COI or 18S) and sediment age group.

**Fig. S5:** Boxplots show variation in (A) Shannon diversity index for COI and (C) for 18S, and (B) ASV richness for COI and (D) for 18S, among biological replicates (cores) across sites and sediment age group for all the metazoan community. Each box represents the distribution of technical replicates for a given biological replicate and sediment age group, overlaid as individual points. Colours indicate site and sediment age group, with lighter shades representing recent sediments and darker shades representing deeper (“old”) horizons.

**Fig. S6:** NMDS ordination including individual PCR replicates (technical replicates) showing increased within-sample dispersion compared to analyses based on averaged community profiles. The plot shows axes 1 and 2 of the two-dimensional NMDS solution based on Bray–Curtis dissimilarities of ASV relative abundances (stress value indicated in the panel). Points are coloured by site (CCO, CLR, STO) and sediment age group (recent vs. old), and shaped according to biological replicate (CoreRep) while shaded ellipses delimit the dispersion of biological replicates (cores) within each site.

**Fig. S7:** Comparison of Bray–Curtis dissimilarities between technical and biological replicates for each marker (COI and 18S). Distributions are shown as boxplots, with the median indicated by a bold horizontal line within each box.

**Fig. S8:** Non-metric multidimensional scaling (NMDS) ordination of COI- and 18S-based metabarcoding-based *seda*DNA Metazoan community composition across the three intertidal sites. The plot shows axes 1 and 2 of the two-dimensional NMDS solution based on Bray–Curtis dissimilarities of ASV relative abundances (stress value indicated in the panel). Points are coloured by site (CCO, CLR, STO) and shaped according to sediment age group (recent vs. old), while shaded ellipses delimit the dispersion of biological replicates (cores) within each site.

**Fig. S9:** Relative contribution of environmental and spatial factors to community structure across datasets. Stacked barplots showing the mean proportion of variance explained by site, sediment age group, and random effects (site and core) across all taxa for each dataset (Eukaryota and Metazoa; COI and 18S markers), based on hierarchical modelling of species communities (jSDM). Values represent posterior means of variance partitioning.

**Fig. S10:** Variance partitioning at the phylum level across datasets. Stacked barplots showing the proportion of variance explained by site, sediment age group, and random effects (site and core) for each phylum across all datasets (Eukaryota and Metazoa; COI and 18S markers), based on hierarchical modelling of species communities (jSDM). Each bar represents an individual phylum. Values represent posterior means of variance partitioning.

**Fig. S11:** Distance-based redundancy analysis (db-RDA) of metabarcoding-based *seda*DNA community composition based on Bray–Curtis dissimilarities. Points represent individual samples coloured by site (CCO, CLR, STO) and shaped by sediment age group (recent vs. old). Ellipses show the 68% dispersion of samples within each site. The ordination is constrained by site and sediment age group, and the first two canonical axes (dbRDA1 and dbRDA2) are shown with their percentage of constrained variation explained. Arrows indicate fitted environmental contrasts derived from the metadata: “recent” versus “old” sediments (4–5 cm vs. 45–50 cm) and “west” versus “east” region (STO vs. CCO/CLR).

**Fig. S12:** Bar plots showing marginal R² values from PERMANOVA models (adonis2, 999 permutations) illustrating the relative contribution of site, sediment age group, and biological replication to Bray–Curtis metazoan community dissimilarities for COI and 18S datasets. Marginal R² values represent the unique contribution of each factor after accounting for the others and should be interpreted comparatively rather than as additive proportions of explained variance.

**Fig. S13:** Sensitivity analysis evaluating the effect of sequencing-depth standardisation on ASV richness estimates. (A) Relationship between observed and rarefied richness for COI and 18S PCR replicates after sample-size rarefaction (10,540 reads for COI and 20,662 reads for 18S). (B) Relationship between rarefied richness and sequencing depth.

**Supplementary tables**

**Table S1:** Sampling locations and core characteristics for metabarcoding-based *seda*DNA analyses. Geographic coordinates (UTM), site names, and total core depths are reported for each sampling location.

| **Core ID** | **Location** | **Latitude** | **Longitude** | **Core depth (cm)** |
| --- | --- | --- | --- | --- |
| **STO** | **Santoña** | **43°26'46.90"N** | **3°27'58.62"W** | **100** |
| **CCO** | **Cicero** | **43°25'01.13"N** | **3°28'40.92"W** | **50** |
| **CLR** | **Colindres** | **43°24'03.02"N** | **3°27'14.80"W** | **50** |

**Table S2:** Primer sequences (5′–3′) used to amplify the COI (Leray-XT) and 18S (V7 region) markers in this study. Primers were dual-indexed using mirrored 8-bp tags to minimise tag-jumping artefacts.

| **Marker** | **Primer name** | **Sequence (5’ → 3’)** | **Reference** |
| --- | --- | --- | --- |
| **COI** | mlCOIintF-XT | GGWACWRGWTGRACWITITAYCCYCC | Wangensteen et al., 2018 |
| **COI** | jgHCO2198 | TAIACYTCIGGRTGICCRAARAAYCA | Wangensteen et al., 2018 |
| **18S** | 18S_allshort_F | TTTGTCTGSTTAATTSCG | Guardiola et al., 2015 |
| **18S** | 18S_allshort_R | TCACAGACCTGTTATTGC | Guardiola et al., 2015 |

**Table S3:** Sediment accumulation rates derived from excess ^210^Pb activities using the “Simple” Model of ^210^Pb dating (Robbins, 1978) for each sediment core. The table shows the estimated sediment accumulation rate (SAR) together with the associated 2σ uncertainty range derived from the regression of ln-transformed excess ^210^Pb activity against depth. The depth at which supported ^210^Pb activity is reached is also indicated.

| **Core** | **Site** | **Depth of supported ^210^Pb (cm)** | **SAR (cm yr^-1^)** | **2σ range** |
| --- | --- | --- | --- | --- |
| STO | Santoña | 10 | 0.1 | - |
| CCO | Cicero | 48 | 0.53 | 0.42 - 0.73 |
| CLR | Colindres | 49 | 0.63 | 0.50 - 0.87 |

**Table S4:** Pearson correlation coefficients (r) for selected lithogenic elements (Fe, Rb, Ti, Zr) between biological replicates within each site, calculated on standardised (z-scored) XRF profiles and restricted to depths shared between cores. Correlation strength is reported descriptively as weak (r < 0.30), moderate (0.30–0.59) and strong (≥ 0.60) to facilitate interpretation.

| **Site** | **Element** | **Core1 vs. Core2 (r)** | **Core1 vs. Core3 (r)** | **Core2 vs. Core3 (r)** | **Correlation** |
| --- | --- | --- | --- | --- | --- |
| **CCO** | **Fe** | **0.293** | **0.038** | **0.005** | **Weak** |
| **CCO** | **Rb** | **0.724** | **0.626** | **0.855** | **Strong** |
| **CCO** | **Ti** | **0.429** | **0.411** | **0.615** | **Moderate** |
| **CCO** | **Zr** | **0.684** | **0.276** | **0.694** | **Strong** |
| **CLR** | **Fe** | **0.661** | **0.558** | **0.800** | **Strong** |
| **CLR** | **Rb** | **0.954** | **0.856** | **0.968** | **Strong** |
| **CLR** | **Ti** | **0.831** | **0.756** | **0.867** | **Strong** |
| **CLR** | **Zr** | **0.747** | **0.264** | **0.836** | **Strong** |
| **STO** | **Fe** | **0.763** | **-0.135** | **0.064** | **Weak** |
| **STO** | **Rb** | **0.286** | **0.425** | **0.848** | **Moderate** |
| **STO** | **Ti** | **0.737** | **0.736** | **0.844** | **Strong** |
| **STO** | **Zr** | **0.488** | **0.346** | **0.824** | **Moderate** |

**Table S5:** Absolute read counts and relative proportions of major taxonomic groups recovered across COI and 18S metabarcoding datasets. Read counts are presented prior to relative-abundance transformation and summarised across all retained sediment samples. Relative proportions indicate the contribution of each major taxonomic group within each marker dataset.

| **Marker** | **Kingdom** | **Total read counts** | **Percentage** |
| --- | --- | --- | --- |
| **COI** | **Unassigned** | **435752** | **53.08** |
|  | **Metazoa** | **200713** | **24.45** |
|  | **Protist** | **93794** | **11.43** |
|  | **Fungi** | **81265** | **9.90** |
|  | **Plantae** | **9350** | **1.14** |
| **18S** | **Unassigned** | **3737679** | **29.9** |
|  | **Metazoa** | **1088146** | **8.70** |
|  | **Protist** | **5070100** | **40.55** |
|  | **Fungi** | **1564356** | **12.51** |
|  | **Plantae** | **1042360** | **8.34** |

| **Community** | **Marker** | **Diversity** | **Variance (Sample)** | **Variance (Residual)** | **ICC** | **Interpretation** |
| --- | --- | --- | --- | --- | --- | --- |
| **Eukariotic** | **COI** | Shannon | 0.477 | 0.212 | 0.69 | High repeatability |
|  |  | Richness | 0.162 |  | 0.29 | Low repeatability |
|  | **18S** | Shannon | 0.197 | 0.156 | 0.56 | Moderate repeatability |
|  |  | Richness | 0.139 |  | 0.31 | Moderate repeatability |
| **Metazoan** | **COI** | Shannon | 0.233 | 0.312 | 0.43 | Moderate repeatability |
|  |  | Richness | 0.091 |  | 0.18 | Low repeatability |
|  | **18S** | Shannon | 0.269 | 0.253 | 0.52 | Moderate repeatability |
|  |  | Richness | 0.105 |  | 0.27 | Low repeatability |

**Table S6:** Variance components and intraclass correlation coefficients (ICC) from mixed-effects models evaluating repeatability among PCR replicates for richness and Shannon diversity. Site and sediment age group were included as fixed effects, while sample identity (Site:Core_Rep:AgeGroup) was included as a random effect. Variance (Sample) corresponds to variance attributable to differences among samples. For Shannon diversity models (lmer), residual variance represents within-sample variability among PCR replicates. For richness models (glmer.nb), residual variance is not reported because negative binomial models do not estimate a separate residual variance component. ICC values were interpreted as follows: >0.6 (high repeatability, low relative technical variation), 0.3–0.6 (moderate repeatability, noticeable technical variation), and <0.3 (low repeatability, substantial technical variation).

**Table S7:** Effects of biological replication (core) on alpha diversity in the complete eukaryotic community. Pairwise comparisons among biological replicates (cores) were performed using linear (Shannon) and negative binomial (richness) models. Core identity (Core_Rep) was included as a fixed effect nested within each Marker × Site × AgeGroup combination. Estimates represent differences between cores (log scale for richness), with standard errors (SE), Wald statistics, and adjusted p-values (Tukey correction). Significant values (p < 0.05) indicate differences among biological replicates within the same marker, site, and sediment age group.

| **Gene** | **Diversity** | **Age Group** | **Site** | **Biological Replicate** | **Estimate** | **SE** | **t ratio** | **P value** |
| --- | --- | --- | --- | --- | --- | --- | --- | --- |
| **COI** | **Shannon** | **Recent** | **CCO** | 1 vs. 2 | -0.614 | 0.214 | -2.87 | 0.580 |
|  |  |  |  | 1 vs. 3 | -0.630 | 0.231 | -2,727 | 0.694 |
|  |  |  |  | 2 vs. 3 | -0.016 | 0.231 | -0.069 | 1.000 |
|  |  |  | **CLR** | 1 vs. 2 | -0.294 | 0.231 | -1.271 | 1.000 |
|  |  |  |  | 1 vs. 3 | -0.309 | 0.231 | -1.338 | 1.000 |
|  |  |  |  | 2 vs. 3 | -0.016 | 0.247 | -0.063 | 1.000 |
|  |  |  | **STO** | 1 vs. 2 | 2.123 | 0.231 | 9.190 | **<0.001*** |
|  |  |  |  | 1 vs. 3 | 1.738 | 0.238 | 7.303 | **<0.001*** |
|  |  |  |  | 2 vs. 3 | -0.385 | 0.221 | -1.739 | 0.999 |
|  |  | **Old** | **CCO** | 1 vs. 2 | -0.237 | 0.238 | -0.994 | 1.000 |
|  |  |  |  | 1 vs. 3 | 0.175 | 0.229 | 0.767 | 1.000 |
|  |  |  |  | 2 vs. 3 | 0.412 | 0.238 | 1.731 | 0.999 |
|  |  |  | **CLR** | 1 vs. 2 | 0.113 | 0.250 | 0.452 | 1.000 |
|  |  |  |  | 1 vs. 3 | 0.408 | 0.250 | 1.628 | 1.000 |
|  |  |  |  | 2 vs. 3 | 0.295 | 0.229 | 1.288 | 1.000 |
|  |  |  | **STO** | 1 vs. 2 | 0.718 | 0.214 | 3.358 | 0.228 |
|  |  |  |  | 1 vs. 3 | 1.893 | 0.214 | 8.852 | **<0.001*** |
|  |  |  |  | 2 vs. 3 | 1.175 | 0.214 | 5.495 | **<0.001*** |
|  | **Richness** | **Recent** | **CCO** | 1 vs. 2 | 0.108 | 0.304 | 0.355 | 1.000 |
|  |  |  |  | 1 vs. 3 | -0.102 | 0.329 | -0.312 | 1.000 |
|  |  |  |  | 2 vs. 3 | -0.210 | 0.329 | -0.640 | 1.000 |
|  |  |  | **CLR** | 1 vs. 2 | -0.189 | 0.329 | -0.574 | 1.000 |
|  |  |  |  | 1 vs. 3 | -0.091 | 0.329 | -0.278 | 1.000 |
|  |  |  |  | 2 vs. 3 | 0.097 | 0.351 | 0.277 | 1.000 |
|  |  |  | **STO** | 1 vs. 2 | 1.457 | 0.329 | 4.431 | **0.005*** |
|  |  |  |  | 1 vs. 3 | 1.014 | 0.339 | 2.993 | 0.473 |
|  |  |  |  | 2 vs. 3 | -0.444 | 0.315 | -1.407 | 1.000 |
|  |  | **Old** | **CCO** | 1 vs. 2 | 0.091 | 0.339 | 0.270 | 1.000 |
|  |  |  |  | 1 vs. 3 | 0.115 | 0.325 | 0.353 | 1.000 |
|  |  |  |  | 2 vs. 3 | 0.023 | 0.339 | 0.069 | 1.000 |
|  |  |  | **CLR** | 1 vs. 2 | -0.623 | 0.357 | -1.745 | 0.999 |
|  |  |  |  | 1 vs. 3 | -0.877 | 0.357 | -2.459 | 0.876 |
|  |  |  |  | 2 vs. 3 | -0.254 | 0.325 | -0.782 | 1.000 |
|  |  |  | **STO** | 1 vs. 2 | 0.776 | 0.306 | 2.535 | 0.833 |
|  |  |  |  | 1 vs. 3 | 1.216 | 0.307 | 3.961 | **0.032*** |
|  |  |  |  | 2 vs. 3 | 0.440 | 0.308 | 1.428 | 1.000 |
| **18S** | **Shannon** | **Recent** | **CCO** | 1 vs. 2 | -0.799 | 0.214 | -3.737 | 0.081 |
|  |  |  |  | 1 vs. 3 | -0.788 | 0.214 | -3.685 | 0.095 |
|  |  |  |  | 2 vs. 3 | 0.011 | 0.214 | 0.052 | 1.000 |
|  |  |  | **CLR** | 1 vs. 2 | -0.273 | 0.214 | -1.274 | 1.000 |
|  |  |  |  | 1 vs. 3 | -0.339 | 0.214 | -1.583 | 1.000 |
|  |  |  |  | 2 vs. 3 | -0.066 | 0.214 | -0.309 | 1.000 |
|  |  |  | **STO** | 1 vs. 2 | -0.194 | 0.214 | -0.908 | 1.000 |
|  |  |  |  | 1 vs. 3 | 1.077 | 0.214 | 5.035 | **<0.001*** |
|  |  |  |  | 2 vs. 3 | 1.271 | 0.214 | 5.943 | **<0.001*** |
|  |  | **Old** | **CCO** | 1 vs. 2 | -0.318 | 0.221 | -1.436 | 1.000 |
|  |  |  |  | 1 vs. 3 | -0.595 | 0.214 | -2.782 | 0.651 |
|  |  |  |  | 2 vs. 3 | -0.277 | 0.221 | -1.251 | 1.000 |
|  |  |  | **CLR** | 1 vs. 2 | 0.402 | 0.214 | 1.879 | 0.996 |
|  |  |  |  | 1 vs. 3 | -0.141 | 0.214 | -0.659 | 1.000 |
|  |  |  |  | 2 vs. 3 | -0.543 | 0.214 | -2.539 | 0825 |
|  |  |  | **STO** | 1 vs. 2 | -0.289 | 0.250 | -1.156 | 1.000 |
|  |  |  |  | 1 vs. 3 | 1.061 | 0.229 | 4.639 | **0.003**** |
|  |  |  |  | 2 vs. 3 | 1.350 | 0.250 | 5.390 | **<0.001*** |
|  | **Richness** | **Recent** | **CCO** | 1 vs. 2 | -0.020 | 0.305 | -0.066 | 1.000 |
|  |  |  |  | 1 vs. 3 | -0.421 | 0.304 | -1.382 | 1.000 |
|  |  |  |  | 2 vs. 3 | -0.401 | 0.304 | -1.316 | 1.000 |
|  |  |  | **CLR** | 1 vs. 2 | 0.363 | 0.304 | 1.192 | 1.000 |
|  |  |  |  | 1 vs. 3 | -0.302 | 0.304 | -0.992 | 1.000 |
|  |  |  |  | 2 vs. 3 | -0.665 | 0.304 | -2.184 | 0.970 |
|  |  |  | **STO** | 1 vs. 2 | -0.093 | 0.304 | -0.306 | 1.000 |
|  |  |  |  | 1 vs. 3 | -0.180 | 0.304 | -0.590 | 1.000 |
|  |  |  |  | 2 vs. 3 | -0.087 | 0.304 | -0.284 | 1.000 |
|  |  | **Old** | **CCO** | 1 vs. 2 | -0.076 | 0.315 | -0.241 | 1.000 |
|  |  |  |  | 1 vs. 3 | -0.404 | 0.304 | -1.330 | 1.000 |
|  |  |  |  | 2 vs. 3 | -0.329 | 0.315 | -1.044 | 1.000 |
|  |  |  | **CLR** | 1 vs. 2 | 0.159 | 0.304 | 0.522 | 1.000 |
|  |  |  |  | 1 vs. 3 | -0.549 | 0.304 | -1.805 | 0.999 |
|  |  |  |  | 2 vs. 3 | -0.708 | 0.304 | -2.327 | 0.932 |
|  |  |  | **STO** | 1 vs. 2 | 0.552 | 0.357 | 1.544 | 1.000 |
|  |  |  |  | 1 vs. 3 | 1.288 | 0.327 | 3.938 | **0.035*** |
|  |  |  |  | 2 vs. 3 | 0.736 | 0.359 | 2.053 | 0.988 |

**Table S8:** Effects of biological replication (core) on alpha diversity in the metazoan community**.** Pairwise comparisons among biological replicates (cores) were performed using linear (Shannon) and negative binomial (richness) models. Core identity (Core_Rep) was included as a fixed effect nested within each Marker × Site × Age Group combination. Estimates represent differences between cores (log scale for richness), with standard errors (SE), Wald statistics, and adjusted p-values (Tukey correction). Significant values (p < 0.05) indicate differences among biological replicates within the same marker, site, and sediment age group.

| **Gene** | **Diversity** | **Age Group** | **Site** | **Biologiocal Replicate** | **Estimate** | **SE** | **t ratio** | **P value** |
| --- | --- | --- | --- | --- | --- | --- | --- | --- |
| **COI** | **Shannon** | **Recent** | **CCO** | 1 vs. 2 | 0.234 | 0.264 | 0.888 | 1.000 |
|  |  |  |  | 1 vs. 3 | -0.745 | 0.285 | -2.614 | 0.776 |
|  |  |  |  | 2 vs. 3 | -0.979 | 0.285 | -3.436 | 0.188 |
|  |  |  | **CLR** | 1 vs. 2 | -0.072 | 0.285 | -0.251 | 1.000 |
|  |  |  |  | 1 vs. 3 | -0.146 | 0.285 | -0.513 | 1.000 |
|  |  |  |  | 2 vs. 3 | -0.075 | 0.304 | -0.245 | 1.000 |
|  |  |  | **STO** | 1 vs. 2 | 0.844 | 0.285 | 2.964 | 0.503 |
|  |  |  |  | 1 vs. 3 | 0.760 | 0.293 | 2.589 | 0.793 |
|  |  |  |  | 2 vs. 3 | -0.085 | 0.273 | -0.310 | 1.000 |
|  |  | **Old** | **CCO** | 1 vs. 2 | 0.034 | 0.293 | 0.117 | 1.000 |
|  |  |  |  | 1 vs. 3 | 0.417 | 0.282 | 1.481 | 1.000 |
|  |  |  |  | 2 vs. 3 | 0.383 | 0.293 | 1.306 | 1.000 |
|  |  |  | **CLR** | 1 vs. 2 | 0.026 | 0.309 | 0.085 | 1.000 |
|  |  |  |  | 1 vs. 3 | -0.848 | 0.309 | -2.744 | 0.681 |
|  |  |  |  | 2 vs. 3 | -0.874 | 0.282 | -3.099 | 0.397 |
|  |  |  | **STO** | 1 vs. 2 | 0.075 | 0.264 | 0.286 | 1.000 |
|  |  |  |  | 1 vs. 3 | 0.212 | 0.264 | 0.804 | 1.000 |
|  |  |  |  | 2 vs. 3 | 0.137 | 0.264 | 0.519 | 1.000 |
|  | **Richness** | **Recent** | **CCO** | 1 vs. 2 | 1.226 | 0.353 | 3.473 | 0.157 |
|  |  |  |  | 1 vs. 3 | 0.419 | 0.342 | 1.223 | 1.000 |
|  |  |  |  | 2 vs. 3 | -0.808 | 0.387 | -2.089 | 0.984 |
|  |  |  | **CLR** | 1 vs. 2 | -0.493 | 0.330 | -1.495 | 1.000 |
|  |  |  |  | 1 vs. 3 | 0.018 | 0.344 | 0.051 | 1.000 |
|  |  |  |  | 2 vs. 3 | 0.511 | 0.355 | 1.439 | 1.000 |
|  |  |  | **STO** | 1 vs. 2 | 0.856 | 0.411 | 2.081 | 0.985 |
|  |  |  |  | 1 vs. 3 | 1.157 | 0.454 | 2.551 | 0.824 |
|  |  |  |  | 2 vs. 3 | 0.302 | 0.469 | 0.643 | 1.000 |
|  |  | **Old** | **CCO** | 1 vs. 2 | -0.116 | 0.346 | -0.337 | 1.000 |
|  |  |  |  | 1 vs. 3 | 0.228 | 0.342 | 0.666 | 1.000 |
|  |  |  |  | 2 vs. 3 | 0.344 | 0.352 | 0.977 | 1.000 |
|  |  |  | **CLR** | 1 vs. 2 | -0.159 | 0.362 | -0.412 | 1.000 |
|  |  |  |  | 1 vs. 3 | -0.429 | 0.356 | -1.204 | 1.000 |
|  |  |  |  | 2 vs. 3 | -0.280 | 0.320 | -0.875 | 1.000 |
|  |  |  | **STO** | 1 vs. 2 | 0.118 | 0.550 | 0.214 | 1.000 |
|  |  |  |  | 1 vs. 3 | 0.405 | 0.586 | 0.692 | 1.000 |
|  |  |  |  | 2 vs. 3 | 0.288 | 0.598 | 0.481 | 1.000 |
| **18S** | **Shannon** | **Recent** | **CCO** | 1 vs. 2 | -0.109 | 0.264 | -0.413 | 1.000 |
|  |  |  |  | 1 vs. 3 | 0.020 | 0.264 | 0.075 | 1.000 |
|  |  |  |  | 2 vs. 3 | 0.129 | 0.264 | 0.488 | 1.000 |
|  |  |  | **CLR** | 1 vs. 2 | -0.784 | 0.264 | -2.975 | 0.494 |
|  |  |  |  | 1 vs. 3 | -0.533 | 0.264 | -2.021 | 0.988 |
|  |  |  |  | 2 vs. 3 | 0.252 | 0.264 | 0.954 | 1.000 |
|  |  |  | **STO** | 1 vs. 2 | 1.195 | 0.264 | 4.532 | **0.005*** |
|  |  |  |  | 1 vs. 3 | 2.035 | 0.264 | 7.715 | **<0.001*** |
|  |  |  |  | 2 vs. 3 | 0.840 | 0.264 | 3.184 | 0.336 |
|  |  | **Old** | **CCO** | 1 vs. 2 | 0.329 | 0.273 | 1.204 | 1.000 |
|  |  |  |  | 1 vs. 3 | 0.074 | 0.264 | 0.280 | 1.000 |
|  |  |  |  | 2 vs. 3 | -0.255 | 0.273 | -0.934 | 1.000 |
|  |  |  | **CLR** | 1 vs. 2 | 0.541 | 0.264 | 2.050 | 0.986 |
|  |  |  |  | 1 vs. 3 | 0.258 | 0.264 | 0.978 | 1.000 |
|  |  |  |  | 2 vs. 3 | -0.283 | 0.264 | -1.072 | 1.000 |
|  |  |  | **STO** | 1 vs. 2 | 0.763 | 0.309 | 2.470 | 0.863 |
|  |  |  |  | 1 vs. 3 | 1.141 | 0.282 | 4.048 | **0.029*** |
|  |  |  |  | 2 vs. 3 | 0.378 | 0.309 | 1.225 | 1.000 |
|  | **Richness** | **Recent** | **CCO** | 1 vs. 2 | 0.098 | 0.263 | 0.371 | 1.000 |
|  |  |  |  | 1 vs. 3 | -0.007 | 0.263 | -0.025 | 1.000 |
|  |  |  |  | 2 vs. 3 | -0.104 | 0.263 | -0.396 | 1.000 |
|  |  |  | **CLR** | 1 vs. 2 | 0.491 | 0.263 | 1.867 | 0.998 |
|  |  |  |  | 1 vs. 3 | -0.136 | 0.261 | -0.520 | 1.000 |
|  |  |  |  | 2 vs. 3 | -0.627 | 0.263 | -2.386 | 0.910 |
|  |  |  | **STO** | 1 vs. 2 | 1.191 | 0.275 | 4.338 | **0.007*** |
|  |  |  |  | 1 vs. 3 | 0.271 | 0.267 | 1.016 | 1.000 |
|  |  |  |  | 2 vs. 3 | -0.921 | 0.276 | -3.336 | 0.226 |
|  |  | **Old** | **CCO** | 1 vs. 2 | 0.162 | 0.280 | 0.578 | 1.000 |
|  |  |  |  | 1 vs. 3 | -0.129 | 0.269 | -0.480 | 1.000 |
|  |  |  |  | 2 vs. 3 | -0.291 | 0.279 | -1.042 | 1.000 |
|  |  |  | **CLR** | 1 vs. 2 | 0.274 | 0.264 | 1.039 | 1.000 |
|  |  |  |  | 1 vs. 3 | -0.345 | 0.262 | -1.319 | 1.000 |
|  |  |  |  | 2 vs. 3 | -0.619 | 0.263 | -2.357 | 0.922 |
|  |  |  | **STO** | 1 vs. 2 | 1.200 | 0.336 | 3.571 | 0.118 |
|  |  |  |  | 1 vs. 3 | 1.169 | 0.304 | 3.850 | **0.048*** |
|  |  |  |  | 2 vs. 3 | -0.031 | 0.349 | -0.090 | 1.000 |

**Table S9:** Effects of site on alpha diversity in the complete eukaryotic community. Pairwise comparisons among sites were performed using mixed-effects models including Site, Marker, and Age Group as fixed effects, with biological replicates (cores) included as a random effect. Estimates represent differences between sites within each Marker × Age Group combination, with standard errors (SE), z-values, and adjusted p-values (Tukey correction).

| **Gene** | **Diversity** | **Age Group** | **Site Intercept** | **Estimate** | **SE** | **t ratio** | **P value** |
| --- | --- | --- | --- | --- | --- | --- | --- |
| **COI** | **Shannon** | **Recent** | CCO-CLR | -0.595 | 0.388 | -1.533 | 0.328 |
|  |  |  | CCO-STO | 0.513 | 0.387 | 1.324 | 0.423 |
|  |  |  | CLR-STO | 1.107 | 0.389 | 2.849 | 0.052 |
|  |  | **Old** | CCO-CLR | 0.753 | 0.390 | 1.930 | 0.193 |
|  |  |  | CCO-STO | 2.059 | 0.387 | 5.326 | **0.002*** |
|  |  |  | CLR-STO | 1.306 | 0.387 | 3.370 | **0.025*** |
|  | **Richness** | **Recent** | CCO-CLR | -0.203 | 0.267 | -0.760 | 0.727 |
|  |  |  | CCO-STO | 0.682 | 0.266 | 2.564 | **0.028*** |
|  |  |  | CLR-STO | 0.885 | 0.270 | 3.281 | **0.003*** |
|  |  | **Old** | CCO-CLR | 0.576 | 0.274 | 2.104 | 0.089 |
|  |  |  | CCO-STO | 2.256 | 0.265 | 8.509 | **< 0.001*** |
|  |  |  | CLR-STO | 1.680 | 0.268 | 6.270 | **< 0.001*** |
| **18S** | **Shannon** | **Recent** | CCO-CLR | -0.772 | 0.384 | -2.011 | 0.176 |
|  |  |  | CCO-STO | -0.089 | 0.384 | -0.231 | 0.971 |
|  |  |  | CLR-STO | 0.683 | 0.384 | 1.779 | 0.240 |
|  |  | **Old** | CCO-CLR | -1.006 | 0.384 | -2.616 | 0.075 |
|  |  |  | CCO-STO | -0.708 | 0.388 | -1.825 | 0.224 |
|  |  |  | CLR-STO | 0.298 | 0.387 | 0.770 | 0.732 |
|  | **Richness** | **Recent** | CCO-CLR | -0.502 | 0.258 | -1.950 | 0.125 |
|  |  |  | CCO-STO | -0.121 | 0.258 | -0.470 | 0.886 |
|  |  |  | CLR-STO | 0.381 | 0.259 | 1.473 | 0.304 |
|  |  | **Old** | CCO-CLR | -0.437 | 0.274 | -1.590 | 0.210 |
|  |  |  | CCO-STO | 1.330 | 0.269 | 4.953 | **< 0.001*** |
|  |  |  | CLR-STO | 1.768 | 0.267 | 6.616 | **< 0.001*** |

**Table S10:** Effects of site on alpha diversity in the metazoan community. Pairwise comparisons among sites were performed using mixed-effects models including Site, Marker, and Age Group as fixed effects, with biological replicates (cores) included as a random effect. Estimates represent differences between sites within each Marker × Age Group combination, with standard errors (SE), z-values, and adjusted p-values (Tukey correction).

| **Gene** | **Diversity** | **Age Group** | **Site Intercept** | **Estimate** | **SE** | **t ratio** | **P value** |
| --- | --- | --- | --- | --- | --- | --- | --- |
| **COI** | **Shannon** | **Recent** | CCO-CLR | 0.591 | 0.308 | 1.921 | 0.180 |
|  |  |  | CCO-STO | 0.019 | 0.306 | 0.061 | 0.998 |
|  |  |  | CLR-STO | -0.573 | 0.309 | -1.854 | 0.198 |
|  |  | **Old** | CCO-CLR | -0.012 | 0.312 | -0.040 | 0.999 |
|  |  |  | CCO-STO | 1.156 | 0.306 | 3.783 | **0.009*** |
|  |  |  | CLR-STO | 1.169 | 0.307 | 3.805 | **0.008*** |
|  | **Richness** | **Recent** | CCO-CLR | -0.375 | 0.281 | -1.335 | 0.376 |
|  |  |  | CCO-STO | 0.799 | 0.301 | 2.657 | **0.022*** |
|  |  |  | CLR-STO | 1.174 | 0.300 | 3.910 | **<0.001*** |
|  |  | **Old** | CCO-CLR | -0.286 | 0.282 | -1.015 | 0.568 |
|  |  |  | CCO-STO | 2.087 | 0.340 | 6.143 | **<0.001*** |
|  |  |  | CLR-STO | 2.373 | 0.339 | 7.000 | **<0.001*** |
| **18S** | **Shannon** | **Recent** | CCO-CLR | -0.390 | 0.301 | -1.295 | 0.430 |
|  |  |  | CCO-STO | 0.032 | 0.301 | 0.107 | 0.994 |
|  |  |  | CLR-STO | 0.422 | 0.301 | 1.403 | 0.377 |
|  |  | **Old** | CCO-CLR | -0.059 | 0.302 | -0.196 | 0.979 |
|  |  |  | CCO-STO | 1.039 | 0.308 | 3.374 | **0.016*** |
|  |  |  | CLR-STO | 1.099 | 0.307 | 3.578 | **0.012*** |
|  | **Richness** | **Recent** | CCO-CLR | -0.242 | 0.250 | -0.971 | 0.595 |
|  |  |  | CCO-STO | 0.705 | 0.252 | 2.803 | **0.014*** |
|  |  |  | CLR-STO | 0.947 | 0.251 | 3.769 | **<0.001*** |
|  |  | **Old** | CCO-CLR | -0.793 | 0.252 | -3.146 | **0.005*** |
|  |  |  | CCO-STO | 0.785 | 0.265 | 2.962 | **0.009*** |
|  |  |  | CLR-STO | 1.578 | 0.263 | 6.007 | **< 0.001*** |

**Table S11:** Mean variance explained by fixed (site, sediment age group) and random effects, alongside model performance metrics (AUC and Tjur’s R²), across all jSDM models. Mean variance partitioning and model performance metrics for hierarchical models of species communities (jSDM), including site, sediment age group, and random effects.

| **Community** | **Marker** | **Site** | **Age Group** | **Random: Site** | **Random: Core** | **Mean AUC** | **Mean Tjur’s R^2^** |
| --- | --- | --- | --- | --- | --- | --- | --- |
| **Eukariotic** | **COI** | 0.669 | 0.213 | 0.060 | 0.059 | 0.941 | 0.234 |
|  | **18S** | 0.766 | 0.140 | 0.049 | 0.045 | 0.929 | 0.277 |
| **Metazoa** | **COI** | 0.630 | 0.283 | 0.046 | 0.040 | 0.959 | 0.263 |
|  | **18S** | 0.740 | 0.164 | 0.047 | 0.049 | 0.935 | 0.220 |

**Table S12:** Results of PERMANOVA (*adonis2*) for all Eukaryotic community and Metazoan community separated between COI and 18S community composition based on Bray–Curtis dissimilarities. The effects of site, sediment age group and biological replicates were tested using marginal permutation tests (999 permutations). R² values represent the proportion of variance explained by each factor, while the residual indicates unexplained variation in community composition not accounted for by the model. Significance codes: *** p < 0.001; ** p < 0.01; * p < 0.05; ns, not significant.

| **Community** | **Marker** | **Factor** | **df** | **R²** | **F** | **p-value** |
| --- | --- | --- | --- | --- | --- | --- |
| **Eukaryotic** | **COI** | **Biological Replicates** | **2** | **0.032** | **0.764** | **0.712** |
|  |  | **Site** | **2** | **0.272** | **3.285** | **0.001** |
|  |  | **Age group** | **1** | **0.157** | **3.797** | **0.001** |
|  |  | **Residual /Unexplained variation** | **11** | **0.539** |  |  |
|  | **18S** | **Biological Replicates** | **2** | **0.029** | **0.825** | **0.516** |
|  |  | **Site** | **2** | **0.345** | **4.977** | **0.001** |
|  |  | **Age group** | **1** | **0.177** | **5.114** | **0.002** |
|  |  | **Residual /Unexplained variation** | **11** | **0.451** |  |  |
| **Metazoan** | **COI** | **Biological Replicates** | **2** | **0.043** | **0.808** | **0.650** |
|  |  | **Site** | **2** | **0.147** | **1.367** | **0.120** |
|  |  | **Age group** | **1** | **0.116** | **2.170** | **0.022** |
|  |  | **Residual /Unexplained variation** | **11** | **0.697** |  |  |
|  | **18S** | **Biological Replicates** | **2** | **0.046** | **0.976** | **0.442** |
|  |  | **Site** | **2** | **0.203** | **3.127** | **0.001** |
|  |  | **Age group** | **1** | **0.133** | **2.785** | **0.001** |
|  |  | **Residual /Unexplained variation** | **11** | **0.619** |  |  |

**Table S13:** Permutation tests for homogeneity of multivariate dispersion (*betadisper*) for eukaryotic and metazoan datasets. The effects of site, sediment age group and biological replicates were tested using marginal permutation tests (999 permutations).

| **Community** | **Gene** | **Factor** | **F** | **p value** |
| --- | --- | --- | --- | --- |
| **Eukaryotic** | **COI** | **Biological Replicate** | 1.384 | 0.263 |
|  |  | **Site** | 7.987 | **0.006** |
|  |  | **Age group** | 12.2 | **0.001** |
|  | **18S** | **Biological Replicate** | 0.082 | 0.919 |
|  |  | **Site** | 1.987 | 0.167 |
|  |  | **Age group** | 43.759 | **0.001** |
| **Metazoan** | **COI** | **Biological Replicate** | 0.335 | 0.691 |
|  |  | **Site** | 0.433 | 0.648 |
|  |  | **Age group** | 1.417 | 0.287 |
|  | **18S** | **Biological Replicate** | 0.318 | 0.736 |
|  |  | **Site** | 1.906 | 0.184 |
|  |  | **Age group** | 1.887 | 0.167 |
